# The Feasibility of Tai Chi Exercise as a Beneficial Mind-Body Intervention in a Group of Community-Dwelling Stroke Survivors with Symptoms of Depression

**DOI:** 10.1155/2021/8600443

**Published:** 2021-11-02

**Authors:** Ruth E. Taylor-Piliae, Helena W. Morrison, Chiu-Hsieh (Paul) Hsu, Susan Whitman, Michael Grandner

**Affiliations:** ^1^College of Nursing, University of Arizona, Tucson, AZ 85721, USA; ^2^College of Public Health, University of Arizona, Tucson, AZ 85724, USA; ^3^College of Medicine, University of Arizona, Tucson, AZ 85724, USA

## Abstract

Depression is prevalent among one-third to two-thirds of acute and chronic stroke survivors. Despite the availability of pharmacotherapies and/or psychotherapies, depression persists, even for 5–10 years after stroke, reflecting limited treatment responses and/or adherence to this conventional care. Mind-body interventions are commonly used among adults to ameliorate depressive symptoms. Thus, the feasibility of Tai Chi, alongside conventional care, to manage poststroke depression was investigated using a single-group pre-post intervention design. Recruitment and retention, intervention adherence, safety, acceptability, and fidelity were assessed. Symptoms of depression, anxiety, and stress were assessed using standardized questionnaires, objective sleep was assessed via a research-grade triaxial accelerometer, and blood samples were taken to measure oxidative stress, inflammatory markers, and a neurotrophic growth factor using commercially available kits per manufacturer's protocol. Pre-post intervention changes were assessed using paired *t*-tests. We enrolled stroke survivors (*N* = 11, mean age = 69.7 ± 9.3) reporting depression symptoms. After the intervention, we observed significant reductions in symptoms of depression (−5.3 ± 5.9, *p*=0.01), anxiety (−2.2 ± 2.4, *p*=0.01), and stress (−4.6 ± 4.8, *p*=0.01), along with better sleep efficiency (+1.8 ± 1.8, *p*=0.01), less wakefulness after sleep onset (−9.3 ± 11.6, *p*=0.04), and less time awake (−9.3 ± 11.6, *p*=0.04). There was a 36% decrease in oxidative stress (*p*=0.02), though no significant changes in the other biomarkers were found (all *p* values >0.05). Tai Chi exercise is a feasible intervention that can be used alongside conventional care to manage poststroke depression, aid in reducing symptoms of anxiety and stress, and improve sleep.

## 1. Introduction

Depression symptoms are widespread among acute and chronic stroke survivors with prevalence rates of 33–66% [1, 2]. Poststroke depression leads to increased disability and mortality rates, along with a higher risk for recurrent stroke [3–5]. Individuals with poststroke depression commonly experience anxiety, stress, and poor sleep [2]. At the biochemical level, stroke survivors with depression have significantly elevated proinflammatory cytokines compared to those without depression [6, 7]. This inflammatory response also disrupts neuroplasticity by decreasing serum brain-derived neurotrophic factor levels, which is predictive of depression [8–10]. While increased levels of oxidative stress are associated with greater depression among stroke survivors [11, 12]. Depression in poststroke patients is currently treated with pharmacotherapies, psychotherapies, or both. Despite the availability of pharmacotherapies and/or psychotherapies, depression persists, even for 5–10 years after stroke, reflecting limited treatment responses and/or adherence to this conventional care [13, 14]. Moreover, the use of pharmacotherapies to treat poststroke depression is associated with adverse events, such as recurrent stroke, seizures, delirium, and dizziness [15].

Mind-body interventions are commonly used among adults to ameliorate depressive symptoms. Tai Chi is a safe and promising mind-body exercise intervention to reduce depressive symptoms [16, 17]. Tai Chi integrates physical movements, breathing training, and mindful awareness [18]. For individuals with depression, Tai Chi provides the practical tools to manage or restructure behaviors and to cultivate autonomy (e.g., choosing to perform Tai Chi), competence (e.g., making progress/mastering Tai Chi movements), and relatedness (e.g., social connections developed through Tai Chi practice) to satisfy their basic psychological needs [19, 20]. In addition, Tai Chi can help sustain and better integrate the connection between mind and body. During Tai Chi practice, it allows the individual to quiet the mind by dwelling in the present and setting aside unnecessary negative emotions. Tai Chi focuses on releasing tension in the body, incorporating mindfulness and imagery into movement, increasing awareness and efficiency of breathing, and promoting overall relaxation of body and mind [21]. Thus, investigating the feasibility of Tai Chi, an established mind-body approach, alongside conventional care to manage poststroke depression is reasonable.

Self-Determination Theory (SDT) was used to guide the delivery of the Tai Chi intervention. SDT has been used for over three decades to direct health behavior change research [22, 23]. In this feasibility study, the intervention was specifically structured to fulfill the three basic psychological needs of autonomy, competence, and relatedness, as outlined in SDT [23, 24]. According to SDT, when these psychological needs are met, individuals benefit from better psychological health, such as less depression [19, 24]. In this study, our overall objective was to determine the feasibility of Tai Chi as a beneficial mind-body intervention, guided by SDT, in a group of community-dwelling stroke survivors with depression.

## 2. Methods

### 2.1. Study Design

In this feasibility study, a single-group pre-post intervention design was used. This study is reported in accordance with the Transparent Reporting of Evaluations with Nonrandomized Designs (TREND) statement [25].

### 2.2. Objectives

The objectives in this study are as follows: (1) determine the feasibility of recruitment and retention, intervention adherence, safety, acceptability, and fidelity of a Tai Chi exercise intervention among community-dwelling stroke survivors with depression and (2) describe changes in symptoms (depression, anxiety, and stress), sleep, and selected biomarkers associated with depression in stroke survivors after the intervention.

### 2.3. Participants

Potential participants were recruited from multiple sources, including flyers (placed at outpatient rehabilitation centers, senior centers, and neurosurgery/neurology offices), and presentations at stroke support groups and outpatient rehabilitation staff meetings. Interested stroke survivors contacted study staff, who screened for eligibility to safely participate, using the Patient Health Questionnaire (PHQ-9) to screen for depressive symptoms. Community-dwelling stroke survivors from all sex/gender, racial/ethnic and socioeconomic groups, aged 55 years and older, at least 3 months after stroke (stable condition after rehabilitation) [26], at least mild depressive symptoms (PHQ 9 > 5) [27], moderate or less disability (Modified Rankin Scale score ≤3) [28], normal cognitive function (Mini-Mental Status Exam score ≥24) [29], and living in the greater Tucson, AZ area were eligible to enroll. Stroke survivors currently practicing Tai Chi greater than once/week, having severe hemineglect, hemianopia, or aphasia [30], a serious psychiatric disorder (e.g., schizophrenia), other serious medical condition [31, 32], or unable to provide informed consent were excluded. Because this is a feasibility study, we did not perform power and sample size calculations based on important or likely differences over time.

### 2.4. Ethical Issues

Approval to conduct the study was obtained from the appropriate Institutional Review Boards. The investigation was carried out according to the principles outlined in the Declaration of Helsinki. Study staff obtained written informed consent from all participants prior to data collection.

### 2.5. Intervention

The Tai Chi intervention protocol (one hour, three times per week for 12-weeks), which was used in our prior research among older adults with cardiovascular disease was implemented [33]. Moreover, this Tai Chi protocol has been used for over 10 years to instruct community-dwelling older adults with/without chronic illness, without safety issues or adverse events. Ms. Edna Silva, RN, an experienced Tai Chi Instructor (>25 years) provided the Tai Chi instruction in this study. The Tai Chi protocol included correct body preparation, standing meditation, and 24 basic movements. Participants were gradually taught the 24 basic movements from the Classic Wu style of Tai Chi (average two new movements per week). Weight shifting was incorporated into all movements, containing one or more of the five basic stances: (a) *parallel* (i.e., stand with feet hip width apart), (b) *empty* (i.e., step forward with one foot placing no weight on it), (c) *T-step* (i.e., turning one foot inward 45–60°), (d) *horse riding* (i.e., stand with feet double width of parallel step), and (e) *archery* (i.e., step forward with one foot, front leg bent with rear leg straight) [34]. During class, participants were asked to replicate the motions, postures, and movement speed of the Tai Chi Instructor. Each movement was broken into its components and practiced in many ways (e.g., using just legs, then just arms, and finally arms and legs together). Each session consisted of a 10-minute warm-up period, 40-minute Tai Chi exercise, and a 10-minute cool-down period. All classes were held at a community-based exercise facility with accessible parking. Each class began with a review of prior content. Participants were standing during classes, but chairs were set up to allow for brief rest periods. Participants were monitored closely for safety (e.g., foot placement) during the class by the Tai Chi Instructor and study staff.

Intervention delivery was guided by SDT, such as allowing participants to make their own decisions and express ideas/opinions (autonomy), learn new skills and instill a sense of accomplishment (competence), and interact with others to create/build meaningful connections (relatedness) [22, 35]. This was accomplished by using noncontrolling language and providing information, encouragement, and feedback within a supportive environment. In addition, the 8 “active ingredients” of Tai Chi as outlined in the Harvard Medical School Guide to Tai Chi [18] were incorporated during class. For example, the instructor describes/corrects the Tai Chi movements to ensure structural integration/correct body posture. In this study, we refined the Tai Chi intervention based on participant feedback and added a brief (5-minute) “circle sharing session” after each class, to allow each person to share how they were feeling, provide comments or ask questions. Each person had the opportunity to share or “pass,” if they wanted. No movements were modified, though due to COVID-19, the Human Subjects Protection Program required us to end the Tai Chi intervention after 8 weeks, instead of the planned 12 weeks.

### 2.6. Outcomes

Study staff built a REDCap database to house all feasibility, symptom, and sleep data collected. Data were collected before and after the intervention by phone and entered directly into REDCap. Before and after the intervention, blood samples were collected by trained personnel and processed for plasma or whole blood and stored (−80°C) in aliquots in the Biological Sciences Laboratory in the College of Nursing at the University of Arizona, to assess selected biomarkers associated with poststroke depression.

### 2.7. Primary Outcomes

#### 2.7.1. Recruitment

Recruitment was assessed as the proportion of respondents who remained interested in the study after information and screening [36]. Our goal was to recruit on average 5 stroke survivors per month for 4 months.

#### 2.7.2. Retention

Retention included the number of participants that completed all aspects of the study (data collection before and after intervention and study intervention), as well as the reasons for attrition [36]. Our goal was at least 80% study retention, with reasons for attrition collected.

#### 2.7.3. Intervention Adherence

Intervention adherence was calculated as the percent of Tai Chi classes attended out of those prescribed [36]. Our goal was at least 80% adherence with reasons for missing the prescribed classes recorded.

#### 2.7.4. Intervention Safety

Intervention safety was assessed as the number of adverse events and serious adverse events occurring during the Tai Chi classes [36, 37]. Our goal was to have no safety issues or adverse events occur.

#### 2.7.5. Intervention Acceptability

Intervention acceptability was assessed by having participants complete a short survey on the acceptability and satisfaction with Tai Chi after intervention [36]. On a scale from 1 to 10, participants were asked to rate their level of intervention acceptability (1 = least acceptable, 10 = most acceptable) and satisfaction (1 = least satisfied, 10 = most satisfied). In addition, they provided yes/no responses to six questions pertaining to classes being offered at a convenient time, difficulty following the instructor, gaining any personal benefit, if their health got better or worse, and if they would recommend the interventions to others. Our goal was at least 75% intervention acceptability.

#### 2.7.6. Intervention Fidelity

Intervention fidelity was assessed using intervention scorecards that were developed for this study, based on the Behavioral Change Consortium's five-component model (i.e., design, training, delivery, receipt, and enactment) [38–40]. Our goal was 90% intervention fidelity using the scorecard.

### 2.8. Secondary Outcomes

#### 2.8.1. Depression

Depression was assessed using the Center for Epidemiological Studies Depression scale (CES-D) [41] and Neuro-QOL (Quality of Life in Neurological Disorders) Depression Short Form (SF) [42]. The CES-D is widely used in research and clinical settings as a screening tool to detect depressive symptoms. The CES-D asks questions pertaining to how the respondent felt or behaved in the past week using a four-point Likert format (0 = none of the time, 3 = most of the time), with possible scores ranging from 0 to 60. Higher scores represent more depressive symptoms. A score of ≥16 using the CES-D is considered a clinical cut-point warranting further evaluation for depression [41]. Construct, convergent, and discriminate validity; high internal consistency; and good test-retest reliability of the CES-D among older adults and stroke survivors have been reported. The Neuro-QOL Depression SF was developed for use among adults living with neurological conditions, such as stroke, and asks how a person felt in the past week. The Neuro-QOL Depression SF contains 8 items, using a five-point Likert format (1 = never, 5 = always), with possible scores ranging from 8 to 40. Higher scores represent more depressive symptoms. Validity and reliability have been reported [43].

#### 2.8.2. Anxiety

Anxiety was assessed using the Generalized Anxiety Disorder Assessment (GAD-7) [44] and the Neuro-QOL Anxiety SF [42]. The GAD-7 is a well-established scale used extensively in research and clinical practice. It contains 7 items and asks a person about problems they may have experienced in the past 2 weeks using a four-point Likert format (0 = not at all, 3 = every day). Possible scores range from 0 to 21, with higher scores indicating greater anxiety (mild = 5–9, moderate = 10–15, severe = >15). Psychometric testing has established construct, convergent, and criterion validity, with high internal consistency reported [44]. The Neuro-QOL Anxiety SF asks how a person felt in the past week. The Neuro-QOL Anxiety SF contains 8 items, using a five-point Likert format (1 = never, 5 = always), with possible scores ranging from 8 to 40. Higher scores represent more anxiety symptoms. Construct validity and high internal consistency have been reported [43].

#### 2.8.3. Stress

Stress was assessed using the Perceived Stress Scale (PSS-10) [45]. The PSS-10 is a widely used, self-administered tool, designed for community samples with limited education. The PSS-10 contains 10 items and asks a person about situations they may have experienced in the past month as being unpredictable, uncontrollable, or overloaded using a five-point Likert format (0 = never, 4 = very often). Possible scores range from 0 to 40, with higher scores indicating greater perceived stress. Psychometric testing has established concurrent, predictive, and known-groups validity with high internal consistency and test-retest reliability [45–47].

#### 2.8.4. Sleep

Sleep was assessed objectively with participants wearing an ActiGraph GT9X Link activity monitor (ActiGraph, Pensacola, FL, USA) on their waist for 1 week before and after intervention [48, 49]. The ActiGraph GT9X Link activity monitor is a research-grade triaxial accelerometer and has been validated to assess sleep in adult populations [48, 49]. The raw data was downloaded using the ActiLife software (version 6.13.4, ActiGraph, Pensacola, FL, USA) and converted into Excel files for use with sleep analysis. The Cole-Kripke sleep algorithm was applied to analyze the data with the Tudor-Locke “default” for sleep period detection. The protocol used in this study was validated in our prior research [50, 51].

#### 2.8.5. Selected Biomarkers Associated with Poststroke Depression

Blood samples were collected from participants and were batch analyzed to measure oxidative stress, inflammatory markers, and a neurotrophic growth factor using commercially available kits per the manufacturer's protocol. At the time of plasma collection, a preservative, butylated hydroxytoluene (0.005% BHT) was added to an aliquot of the plasma sample. Positive controls were used (e.g., spike in known protein concentrations) to ensure assay validity. All data were reported in pg/mL for statistical analysis.

*(1) Oxidative Stress*. Oxidative stress was operationalized by measuring plasma superoxide dismutase (SOD) activity and 8-iso prostaglandin F_2*α*_ (8-isoprostane). SOD was measured using a colorimetric assay (Abcam, ab65354, Cambridge, MA) that assessed the inhibition activity of SOD in a sample. Samples were thawed on ice and the SOD assay was used according to manufacturer instructions. All samples were run in duplicate with an average coefficient of variation (CV) of 1.8%. All data are reported as a percent inhibition, reflective of SOD activity. A competitive ELISA assay (Cayman Chemical cat516351, Ann Arbor, MI) was used to measure plasma 8-isoprostane, a biologically active chemical and marker of oxidative stress. The BHT preserved plasma samples were stored at −80°C prior to use, and samples were thawed on ice prior to use. The assay was used according to manufacturer instructions and the range was reported as 0.8–500 pg/ml and sensitivity (80% B/B_0_) of 3pg/ml. Plasma samples were purified using a solid-phase extraction cartridge (Caymen #400020) and N_2_ gas according to manufacturer instructions and the assay was used according to manufacturer instructions with no deviations. All samples were run in duplicate with an average CV of 2.4%.

*(2) Inflammatory Markers and Neurotrophic Factor*. Concentrations of inflammatory markers tumor necrosis factor-alpha (TNF-*α*), interleukin- (IL-) 6, IL-10, and brain-derived neurotrophic factor (BDNF) were measured from serum samples. To measure TNF-*α*, IL-6, and IL-10, a serum aliquot was shipped on dry ice to Quanterix (Billerica, MA) for data collection using the Simoa® platform. The Simoa® platform was chosen because of its documented sensitivity to measure low concentrations of analytes [52]. Detection ranges for analytes were as follows: TNF-*α* (0∼100pg/mL), IL-6 (0∼140pg/mL), and IL-10 (0–50pg/mL). All samples were run in duplicate with an average CV of 10%. A Luminex platform was used to measure BDNF in serum samples using an assay kit (R&D Systems LXSAHM-0, Minneapolis, MN). All samples were run in duplicate with an average CV of 4%.

### 2.9. Statistical Methods

Descriptive statistics were calculated for all variables to ensure data quality (check distributions, examine outliers) and describe the sample. To determine recruitment and retention rates, along with intervention adherence, safety, acceptability, and fidelity, descriptive statistics were used and were reported as frequencies and percentages or mean ± SD. To describe changes in symptoms (depression, anxiety, and stress), sleep, and selected biomarkers between baseline and postintervention, paired *t*-tests and signed-rank tests were used. Data were analyzed using SAS statistical software (version 9.4, SAS Institute, Cary, NC, USA).

## 3. Results

### 3.1. Study Enrollment

**Figure 1:**
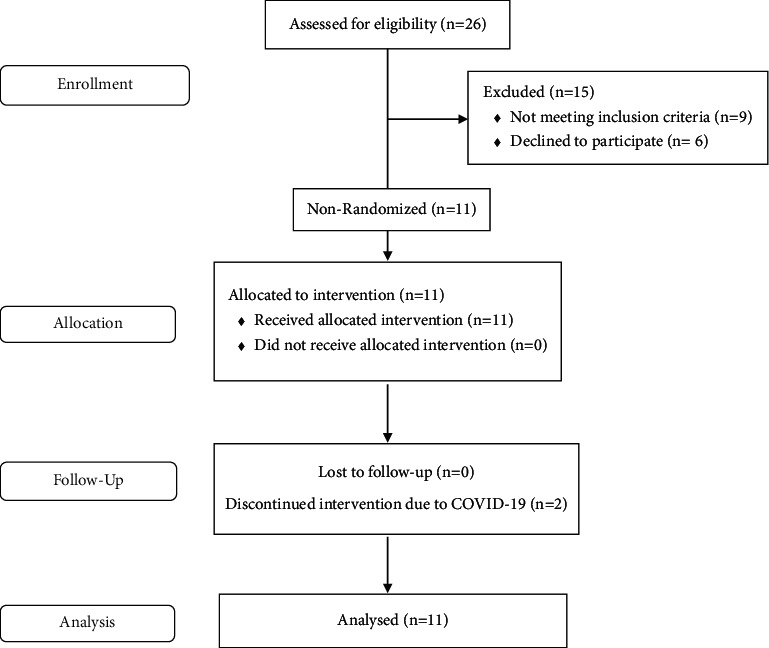
Trend flow diagram.

A total of 26 stroke survivors were assessed for study eligibility, of which 9 did not meet the eligibility criteria and 6 declined study participation. A total of 11 stroke survivors were enrolled in the study. The flow of participants in the study including enrollment, group allocation, follow-up, and analysis is presented in Figure 1.

### 3.2. Description of Protocol Deviation

Due to COVID-19, the Human Subjects Protection Program required us to suspend further study recruitment efforts and to end the ongoing Tai Chi intervention after 8 weeks, instead of the planned 12 weeks.

### 3.3. Recruitment Dates and Follow-Up

Participants were recruited for over 4 months from August to December 2019. Baseline data were collected in January 2020 with postintervention data collected in March 2020, prior to COVID-19 closures.

### 3.4. Baseline Demographic and Clinical Characteristics

Participants were on average 70 years old, mainly retired (73%, *n* = 8), married men (55%, *n* = 6) with >13 years of education (91%, *n* = 10), reporting depression symptoms (CESD = 17.3 ± 11.4) with 55% taking antidepressant medications. The majority of participants reported having an ischemic stroke (82%, *n* = 9) with hemiparesis (55%, *n* = 6), but were able to walk 15 feet without assistance (91%, *n* = 10). Participants self-reported medical history included hypertension (64%, *n* = 7), arthritis (64%, *n* = 7), and prior cancer (36%, *n* = 4) (Table 1).

### 3.5. Primary Outcomes

#### 3.5.1. Recruitment and Retention

Due to COVID-19, the Human Subjects Protection Program required us to suspend study recruitment efforts, and we were unable to meet our recruitment goal of enrolling 20 stroke survivors in the study. While a total of 17 stroke survivors were screened and met the eligibility criteria, 6 declined study participation, with an average recruitment rate of 2.8 participants/month over 4 months. Study retention was 100% (*n* = 11), with all participants completing all aspects of the study (i.e., data collection pre-post intervention and study intervention).

#### 3.5.2. Intervention Acceptability, Safety, Adherence, and Fidelity

Participants' acceptability and satisfaction with the Tai Chi intervention were very high (Table 2). All participants (100%, *n* = 11) reported that the interventions were conducted at a convenient time that they gained personal benefits. Almost all (91%, *n* = 10) would recommend this intervention to others and felt that their health improved. There were no safety issues or adverse events during any of the Tai Chi classes. Participants had very high intervention adherence, attending on average 88% of scheduled classes, with 91% (*n* = 10) of participants attending ≥80% of the scheduled classes. The most frequent reasons for missing class were due to doctor's appointments (*n* = 8), feeling unwell (*n* = 7), or being out of town travel (*n* = 5). Using a scorecard, intervention fidelity was 90%, which was due to ending the Tai Chi intervention early after 8 weeks, as a result of the COVID-19 restrictions.

### 3.6. Secondary Outcomes

#### 3.6.1. Changes in Symptoms of Depression, Anxiety, and Stress

At baseline, participants on average reported mild to moderate symptoms of depression (CES-D = 17.3 ± 11.4), anxiety (GAD-7 = 5.5 ± 4.8), and stress (PSS = 14.3 ± 7.6). After the Tai Chi intervention, we observed statistically significant changes in these symptoms, with less depression, anxiety, and stress reported (Table 3).

#### 3.6.2. Changes in Objective Sleep

Participants were asked to wear an actigraph for 1 week before and after the intervention. Before the intervention, 91% of participants wore the actigraph (*n* = 10), with the majority (*n* = 6) wearing the actigraph for all seven days. Two participants wore the actigraph for 6 days including weekends, and one participant wore the actigraph for 5 days, excluding the weekend.

After the intervention, 82% of participants (*n* = 9) wore the actigraph, with the majority (*n* = 8) wearing the actigraph for all seven days, and one participant wearing the actigraph for 6 days including the weekend. After the Tai Chi intervention, we observed statistically significant changes in sleep with better sleep efficiency (+1.8 ± 1.8, *p*=0.01), less wakefulness after sleep onset (−9.3 ± 11.6, *p*=0.04), and less time awake (−9.3 ± 11.6, *p*=0.04) (Table 3).

#### 3.6.3. Changes in Biomarkers Associated with Poststroke Depression

**Figure 2:**
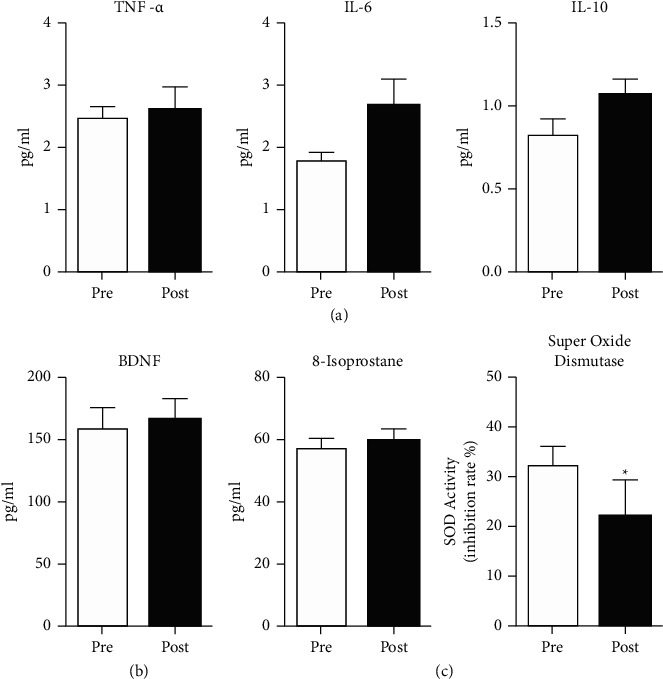
Changes in biomarkers associated with poststroke depression. Summary data of changes in serum biochemicals after Tai Chi intervention include inflammatory markers TNF-*α*, IL-6, and IL-10 (a), neurotrophic factor BDNF (b), and markers of oxidative stress 8-isoprostane and superoxide dismutase (c). Superoxide dismutase was significantly decreased after Tai Chi intervention (^*∗*^*p* < 0.05). Sample size = 9 for all biochemical assays with statistical analysis using paired *t*-test.

After the intervention, 82% of participants (*n* = 9) provided blood samples. We found a 36% decrease in SOD activity (ES = 0.75, *p*=0.02) indicative of a decreased oxidative environment after intervention (Figure 2), though no significant changes in 8-isoprostane were observed. In addition, increases in IL-6 (ES = 0.59) and IL-10 (ES = 0.61) were observed, though these did not reach statistical significance due to the sample size. No significant changes in TNF-*α* or BDNF were found (all *p* values >0.05) (Figure 2).

## 4. Discussion

Among community-dwelling stroke survivors, Tai Chi exercise is a feasible intervention that can be used alongside conventional care to manage poststroke depression and may also aid in reducing symptoms of anxiety and stress and improve sleep. Recruitment of community-dwelling stroke survivors to participate in intervention studies is challenging, particularly among those with poststroke depression [53]. In this study, recruitment was impacted by COVID-19 closures, though our recruitment rates were similar to a recent intervention study to ameliorate poststroke depression [54]. Few studies have examined the effect of Tai Chi on poststroke depression [36, 37, 55], though they reported high study retention rates ranging from 85 to 90%, though less than the 100% retention in this study.

This study is the first to report on all four attributes of Tai Chi exercise feasibility among stroke survivors, namely, the intervention adherence, safety, acceptability, and fidelity. Intervention adherence in this study was high (88%), and similar to prior Tai Chi studies that examined the effect of Tai Chi on poststroke depression (85–92%) [36, 37]. Tai Chi is a safe form of exercise for stroke survivors, including those with hemiparesis or poststroke depression [36, 37, 56], though intervention safety or adverse events are frequently not reported [57–59]. While stroke survivors typically report great enjoyment and satisfaction when performing Tai Chi [37, 56, 60], the acceptability or fidelity of Tai Chi interventions among stroke survivors are frequently not reported [58, 59].

After the Tai Chi intervention, we observed significant reductions in symptoms of depression, anxiety, and stress, along with better sleep. Among stroke survivors, prior studies have examined the effect of Tai Chi on symptoms of depression [36, 37, 55], but the effect of Tai Chi on symptoms of anxiety and stress was not reported. Our findings are consistent with prior studies, reporting less depression among stroke survivors after a Tai Chi intervention [37, 55] and a recent meta-analysis [61], though are in contrast to a Tai Chi intervention study among community-dwelling stroke survivors [36]. While depressive symptoms were assessed in that study [36], the primary outcome was an improvement in physical function and not all stroke survivors had symptoms of depression at baseline. Among stroke survivors, prior studies examining the effect of Tai Chi on sleep are limited and have primarily assessed subjective sleep quality [55].

After the Tai Chi intervention, we observed improvements in several biomarkers associated with poststroke depression. Several clinical trials have reported that the practice of Tai Chi has an antioxidant effect [62]. SOD is an important antioxidant enzyme, which catalyzes the dismutation of the superoxide anion into hydrogen peroxide and molecular oxygen. In this feasibility study, we found a 36% decrease in SOD activity indicating a decreased oxidative environment after the intervention. However, this finding is in contrast to a recent systematic review and meta-analysis [62], reporting an increase in SOD activity following a Tai Chi intervention. Our measurement of SOD quantifies enzyme activity, rather than absolute SOD concentrations. As a single measure of oxidative stress, our findings may be interpreted broadly and possibly in multiple ways. Traditionally and without a broader picture, elevated SOD activity may be interpreted as a biomarker of a low oxidative stress environment because of the consistent and constant conversion of reactive oxygen species to hydrogen peroxide and then to water by catalase and glutathione peroxidase [62, 63]. On the other hand, low SOD activity may result from a reduced oxidative stress environment (e.g., low demand results in low activity), less efficient enzyme activity that is often associated with age, or damage to SOD due to persistent or high levels of oxidative stress [64]. In the future and because determining the physiologic meaning of data based on a single measure is difficult, a more complete picture of the effects of Tai Chi on the oxidative stress environment could be obtained through multiple measures, for example by including measures of catalase and glutathione as well as SOD concentrations [63]. While we also collected data on 8-isoprostane, a biologically active chemical that results from oxidation of arachidonic acid by, for example, hydrogen peroxide, it is considered a quantitative index of lipid peroxidation [65]. In this dataset, SOD activity was significantly decreased; however, 8-isoprostane concentrations were unchanged. Therefore, it is difficult to elucidate the physiologic significance of the findings in this feasibility study.

Moreover, others have reported that the effects of exercise on oxidative stress (e.g., SOD activity) depends on several factors, including the exercise type (aerobic, nonaerobic), intensity (mild, moderate, vigorous), and frequency (sessions per week), as well as an individual's personal characteristics, such as age, gender, exercise capacity, and chronic health conditions [66, 67]. In this study, participants were, on average, 70-year-old stroke survivors with symptoms of depression. Also, there are different sampling methods (blood, saliva, urine) and indicators of oxidative stress (e.g., SOD, *F*_2*α*_ 8-isoprostane, catalase, and glutathione peroxidase) each with unique validation, stability, and reproducibility considerations [68], which may, in part, explain the differences in the findings obtained.

Several studies indicate that increased levels of oxidative stress and inflammatory biomarkers are common among persons with depression [69, 70]. Our findings are consistent with a prior systematic review examining the effect of Tai Chi on inflammatory markers [71], with mixed results found after the intervention. In this study, we observed increases in IL-6 (ES = 0.59) and IL-10 (ES = 0.61) though these did not reach statistical significance due to the sample size, while no significant changes in TNF-*α* or BDNF were found (all *p* values >0.05). IL-6 is a commonly investigated protein and a well-known biomarker of inflammation, stress, and depression, all closely interrelated, in both preclinical and clinical studies [72]. Moreover, IL-6 levels correlate differently among different depression subtypes, symptomology, and may be confounded by chronic inflammatory conditions that are often comorbid with ischemic stroke. Complicating our interpretation of IL-6 biomarker data further, prior research indicates that IL-6 exerts both pro- and anti-inflammatory properties [73, 74]. While IL-6 is generally regarded as having proinflammatory properties, research indicates it has many anti-inflammatory functions as well. This dichotomy of IL-6 functions indicates that it may be responsible for maintaining the balance between pro- and anti-inflammatory responses [73, 74]. Considering these limitations, higher levels of IL-6 have been observed among those with treatment-resistant depression, and among stroke survivors with depression [75, 76].

While IL-10 is studied less frequently than IL-6, others illustrate that IL-10 concentrations are also increased in patients with depression, compared to healthy controls [77] and similar to IL-6, they may be confounded by underlying inflammation and/or stress disorders. IL-10 may be produced by B-cells and Th2 cells of the adaptive immune system. Although IL-10 is broadly considered an “anti-inflammatory” cytokine, it also has known immunosuppressive and immunostimulatory effects that are disease dependent [78]. When considering serum or plasma biomarkers of neurological-based diseases such as IL-6 and IL-10, it must be acknowledged that serum or plasma cytokine levels may not reflect the brain microenvironment, but rather, might be overwhelmed by the systemic milieu [79]. On the other hand, when considering poststroke depression and recent evidence of a leaky glial scar during poststroke recovery in preclinical models [80–82], the brain and systemic milieu may be more intermingled than once appreciated. Despite prior research evidence linking depression, oxidative stress, and inflammatory biomarkers, many aspects remain to be explored in future larger studies with more rigorous study designs, and carefully chosen outcome measures that assess the mechanisms as well as the effects of Tai Chi on these biomarkers associated with poststroke depression.

### 4.1. Study Limitations

Since this was a feasibility study, several limitations should be noted. In this study, our primary outcome was to determine the feasibility of recruitment and retention, intervention adherence, safety, acceptability, and fidelity of a Tai Chi exercise intervention among community-dwelling stroke survivors with depression. A single-group pre-post intervention design was used, which was not powered to assess intervention effectiveness and there was no control/comparison group. While our secondary outcomes used standardized questionnaires to assess changes in symptoms of depression, anxiety, and stress; this self-report data may be subject to recall bias or socially desirable responses. Moreover, the changes in these symptoms may be due to the participants' poststroke recovery improvements. Also, the small sample size limits the generalizability of the results obtained from our objective measures of sleep and biomarkers associated with poststroke depression. Nevertheless, our results support the feasibility and acceptability of Tai Chi exercise for poststroke depression and provide useful information for developing a future large-scale trial.

## 5. Conclusions

Symptoms of depression, anxiety, and stress were observed among these community-dwelling stroke survivors along with suboptimal sleep. Among community-dwelling stroke survivors, Tai Chi exercise is a feasible intervention that can be used alongside conventional care to manage poststroke depression and may also aid in reducing symptoms of anxiety and stress and improve sleep. Further research is needed with rigorous study designs and larger samples before widespread recommendations can be made.

## Figures and Tables

**Table 1:** Participant demographic and clinical characteristics (*n* = 11).

Variable	Mean ± SD or frequency (%)
Age	70.00 ± 9.30
BMI	25.45 ± 4.78
Males	6 (54.55%)
Education	
High school/GED	1 (9.09%)
Some college	3 (27.27%)
Bachelor	4 (36.36%)
Graduate	3 (27.27%)
Employment	
Retired	8 (72.73%)
Disabled	3 (27.27%)
Income	
<16K	3 (27.27%)
25∼50K	3 (27.27%)
50∼75K	1 (9.09%)
>75K	3 (27.27%)
Refused	1 (9.09%)
Race	
White	10 (90.91%)
Latino	1 (9.09%)
Marital status	
Married	6 (54.55%)
Widowed/divorced/never married	5 (45.45%)
Stroke type	
Hemorrhagic	2 (18.18%)
Ischemic	9 (81.82%)
Completed stroke rehabilitation	8 (72.73%)
Hemiparesis	6 (54.55%)
Walk 15 feet without assistance	10 (90.91%)
Uses assistive device	3 (27.27%)
Chronic conditions	
Arthritis	7 (63.64%)
Diabetes	1 (9.09%)
Heart disease	2 (18.18%)
High blood pressure	7 (63.64%)
Cancer	4 (36.36%)
Lung disease	1 (9.09%)
Kidney disease	2 (18.18%)
Tobacco use	1 (9.09%)
Antidepressant medication use	6 (54.55%)

**Table 2:** Intervention acceptability, satisfaction, safety, and adherence (*N* = 11).

Variable	Mean ± SD or frequency (%)
Acceptability^*∗*^, mean ± SD	9.5 ± 0.97 (range: 7 to 10)
Satisfaction^*∗*^, mean ± SD	9.0 ± 1.67 (range: 5 to 10)
Convenient time (%)	11 (100%)
Any difficulty (%)	4 (36.36%)
Gain benefits (%)	11 (100%)
Better health (%)	10 (90.91%)
Worse health (%)	0 (0%)
Recommend to others (%)	10 (90.91%)
Safety/adverse events (%)	0% (*n* = 0)
Adherence/class attendance (%)	88 ± 10.5 (range: 64–100%)

^*∗*^Possible score range = 1–10 (1 = least, 10 = most).

**Table 3:** Summary of symptoms and objective sleep by time point.

Variable	Time 1 (*N* = 11) mean ± SD	Time 2 (*N* = 11) mean ± SD	Time 2–Time 1 (*N* = 11) mean ± SD	*p* value^*∗*^
Depression				
CES-D	17.27 ± 11.42	12.00 ± 8.51	−5.27 ± 5.92	**0.01 (0.03)**
Neuro-QOL depression SF	14.09 ± 7.73	10.82 ± 3.57	−3.27 ± 6.33	0.12 (0.11)

Anxiety				
GAD-7	5.45 ± 4.78	3.27 ± 4.29	−2.18 ± 2.40	**0.01 (0.02)**
Neuro-QOL anxiety SF	14.55 ± 6.89	10.45 ± 4.01	−4.09 ± 4.68	**0.02 (0.02)**

Stress				
PSS	14.27 ± 7.55	9.64 ± 7.90	−4.64 ± 4.76	**0.01 (0.01)**

Objective sleep^#^	Time 1 (*N* = 10)	Time 2 (*N* = 9)	Time 2–Time 1 (*N* = 9)	
Efficiency	93.67 ± 1.89	95.35 ± 2.38	1.81 ± 1.76	**0.01 (0.02)**
TST	8.87 ± 2.08	9.26 ± 2.36	0.52 ± 2.73	0.58 (0.65)
WASO	29.48 ± 8.58	21.00 ± 8.12	−9.34 ± 11.58	**0.04 (0.04)**
Number of awakenings	9.12 ± 3.91	6.94 ± 2.91	−2.55 ± 3.41	0.06 (0.05)
Length of awake time (minutes)	29.48 ± 8.58	21.00 ± 8.12	−9.34 ± 11.58	**0.04 (0.04)**

^*∗*^Derived from paired *t*-test (signed-rank test), ^#^derived from actigraph using the Cole–Kripke analysis method [83]. CES-D = Center for Epidemiological Studies Depression Scale; GAD-7 = Generalized Anxiety Disorder 7-item Scale; PSS = Perceived Stress Scale; SF = Short Form; Bold shows statistically significant p-value, derived from paired t-test (signed-rank test). sleep efficiency = total sleep time/time in bed × 100; TST = total sleep time; WASO = wake after sleep onset; time 1 = preintervention; time 2 = postintervention.

## Data Availability

The data used to support the findings of this study are available from the corresponding author upon request.
